# Healthy Buildings, a Webinar Report

**DOI:** 10.1177/19375867231223885

**Published:** 2024-01-23

**Authors:** Priya Rachel Boby, Laura Cambra-Rufino, Prabhjot Sugga, Anil Dewan

**Affiliations:** 1 Queensland University of Technology, Adelaide, Australia; 2 “Universidad Politécnica de Madrid,” Spain; 3 School of Planning and Architecture, New Delhi, India

**Keywords:** healthy building, webinar, pandemic resilient, infection free spaces, healthcare

The need for a change in the built environment was stressed by the threat of the COVID-19 pandemic coupled with the effects of climate change (Emmanuel et al., 2020; Priyadarsini et al., 2020). According to EPA (2021), humans spend approximately 90% of their time indoors, and spatial qualities can affect physical and mental well-being. Therefore, health-related decisions are made in architectural, spatial, internal facilities, premise plumbing systems, and heating, ventilating, and air-conditioning design (Salonen et al., 2023). Hence, today it is common to speak about “healthy buildings,” defined as “built structures that promote the positive well-being of individuals” (Awada et al., 2021).

Despite the rapid need for architectural solutions during the pandemic, there was not much time for the valuable exchange of knowledge or validation from experts in various countries on this issue. However, architects are expected to implement design solutions for issues highlighted during the COVID-19 pandemic that could not be addressed at that time (Emmanuel et al., 2020).

Hence, the aim of this report is to summarize the key findings from the webinar on healthy buildings, which tackles design aspects driven by the COVID-19 pandemic, which are applicable to healthcare, educational, and commercial buildings in order to reach a larger audience who could benefit from them.

## The Webinar

On November 30, 2022, the School of Planning and Architecture, New Delhi, organized a webinar to offer the students and faculty an opportunity to comprehend the latest design solutions to incorporate into their buildings for well-being. This webinar consisted of nine plenary sessions and a focus group.

### Plenary Sessions

For the plenary session, each speaker had 30–45 min to discuss a topic of their expertise and to conduct a discussion session with the audience at the end. At 1 p.m. IST, Prof. Dr. P. S. N. Rao, Director of the School of Planning and Architecture, New Delhi, and Prof. Dr. Anil Dewan, Head and Professor of the Architecture Department School of Planning and Architecture, New Delhi, launched the sessions. The director described the importance of healthy space and emphasized it as the need of the hour.

### Healthcare Systems

Healthcare systems have undergone intensive modifications, changes, development, and improvement over the last 30 years. The reformation of global healthcare systems has been due to governmental actors, nongovernmental actors, and countries. However, in developing countries, numerous challenges remain (Durrani, 2016).

An examination of the environmental determinants for health revealed that only 6% is based on medical care, even though countries such as the United States spend huge amounts on healthcare, remarked Mr. Sanjit Roy, Assistant Professor at the School of Planning and Architecture, South Illinois University. A participant raised the following concern: “If the U.S. healthcare system is flawed, which country has the best healthcare system?” Mr. Sanjit remarked “Scandinavian countries have the best healthcare systems.”

On the other hand, in India, the rural healthcare system, the Bhore healthcare system, was implemented as highlighted by Prabhjot Sugga, Associate Professor at the School of Planning and Architecture, New Delhi:

In 1946, Mayur Bhanu introduced the Bhore healthcare system as the hierarchy of the healthcare framework for the rural health system. The district healthcare system has primary, secondary, and tertiary units. In fact, Western nations, such as the United States and the United Kingdom, developed a framework for population as 10 per 1,000, number and types of personnel, activities offered, duration of activity, and population density (Bhore committee, 1946).

India is a country with numerous people living in villages; therefore, there is a need for an intensive approach to rural health (Jaysawal, 2015). Dr. Prabhjot Sugga, Associate Professor at the School of Planning and Architecture, New Delhi, noted: The first dispensaries were built by colonialists and were funded by the government. It had high ceilings, thick walls, gable roofs, and a veranda all around. The Ranganatha Primary Health care Centre building in India has a courtyard form for flood resilience and a segregated building for infectious diseases. It has a brick facade and a gable roof with wooden trusses and uses local labor for maintenance. This multilaterally funded (World Bank) building had an integrated ward with surgery and labor rooms. The multistory design allows for future expansion, while the reinforced concrete frame provides strength and stability. The subcenters served a population of 5,000 people, were close to the Panchayat, and were safe at night.

Independent blocks for primary healthcare on high plinths reduce the spread of infections and are flood resilient. The orientation of blocks follows the site form, with a water well for potable water and soak pits for sewerage disposal. The materials used were brick, a wooden truss with asbestos sheeting, and a reinforced concrete frame.

The old rural healthcare infrastructure had thick walls and parapet walls and a good ambiance. The old buildings had courtyards and septic tanks with power backup for self-sustainment.

Prof. Anil Dewan spoke of health facilities built in Haryana by the World Bank. In fact, facilities without a doctor are misused or unused because the public does not trust healthcare workers because they are less qualified than doctors.

### Sanatoriums as a Response to Infectious Diseases

Sanatoriums were built in the 19th and early 20th centuries to treat tuberculosis patients and were a crucial healthcare infrastructure at the time. In fact, studies depict that the Paimio Sanatorium may offer guidance on changes in architectural design post COVID-19 pandemic (Sirel, 2021).

Mr. Sanjit Roy, observed that infectious diseases such as tuberculosis led to the introduction of sanatoriums, which was an innovation by Robert Kuch. Notably, the admittance of sunlight in buildings, fresh air, and rest can speed up a patient’s recovery. In response, Central Park in New York, United States and Hyde Park in London, United Kingdom, were introduced to avoid overcrowded spaces that could spread infections. The Paimio Sanatorium in Finland, built in 1930 by Alvar Alto, is considered a masterpiece for its hygienic surfaces and design elements from historic to present times. In fact, dirt was seen as an “enemy” in this design.

### Link Between Nature and Patient Recovery

Bedrooms with a view to nature have improved physical and mental health in a residential rehabilitation program, even though the degree of change varied with gender and diagnostic group (Raanaas et al., 2012). As Dr. Prabhjot Sugga noted that in fact, research has shown a correlation between patient treatment setting and recovery rate. The recovery rate can be promoted through art, nature, and the humanities. For instance, the pediatric ward in Rairangpur has artwork on its walls and a children’s park with swings and slides.

According to Roger Ulrich, a Professor of Architecture, in 1984, “patients in hospital rooms with windows recovered at a faster rate and in greater percentage than the patients in windowless rooms,” remarked Mr. Sanjay Prakash. Prof. Chandrashekar, former Chief Architect Ministry of Health Visiting faculty at London South Bank, supported the concept and observed that the designs should follow an evidence-based design that states that patients should connect with nature by proximity to a window to speed up recovery rates.

On the other hand, Mr. Sanjay Prakash, Visiting Faculty, School of Planning and Architecture, New Delhi, founder and lead architect at Shift Habitat Studio, pointed out that modern hospitals seem to be boxes into which hundreds of people go with different diseases. Renaissance architects designed these with natural daylight and ventilation. With antibiotics and improved aseptic practices in the 1950s, doctors began to believe that patient health could be maintained regardless of the room design. However, the COVID-19 pandemic has shown us that we should perhaps have something less congested. The Austrian philosopher Ivan Illich argued that “a major threat to health in the world was modern medicine and that hospitals caused more sickness than health.” He popularized the word iatrogenesis to describe what he viewed as an increase in good hospitals that do not necessarily contribute to good health outcomes.

### Indian Design Standards for Hospitals

The Indian healthcare system consists of numerous hospitals of varying sizes and units managed by central and state governments (Gopal, 2019). Meanwhile, the patient care system is distributed through primary/community healthcare, secondary healthcare (district hospital) and tertiary healthcare centers (national level; Bagchi, 2008; Deodhar, 1982).

Prof. Anil Dewan remarked: The Indian hospital standards include the Scales of Accommodation, the Central Public Works Department (CPWD) for the central government, the Bureau of Indian Standards, and the Indian Public Health Standards (IPHS). The standards state that corridors for patients and the public should be segregated within the building with a minimum width of 3 m. In addition, the public areas in a hospital, such as the admission, outpatient department, pharmacy, emergency, corridors, waiting areas, and toilets, require two exits with 2 m width. The dimensions between beds should be 2 m to reduce infections with one sanitary point per 15 beds. In addition, at least 10% of the site area should be used for temporary expansion. Finally, the corridors should be single loaded and shorter with a view to courtyard spaces. However, studies depict that while 33% of designs use the same corridor and entrance, only 11% prefer dividing corridors as clean and dirty pathways. Separate passages are required for entry into the waiting and treatment zones. Moreover, decontamination areas must be provided for ambulances.

Mr. Sanjay Prakash noted: The IPHS allow the layout to vary according to the location and shape of the site, the level of the site, and climatic conditions (IPHS, 2022). These IPHS documents provide sample layouts for various levels of facility, and these tend to become the standardized design used by government agencies and replicated all over the country.

## Materials

Indirect contact infections can be prevented by touchless technologies and the application of antibacterial coatings on regularly touched surfaces (Salonen et al., 2023).

Prof. Dr. Chandrashekhar R. observed that copper is an antimicrobial material and should be used because of its efficacy against pathogens, eliminating more than 99% of the bacteria after 2 hr of exposure. However, the deterrent to using copper is its high cost. Nevertheless, the cost can be reduced using a copper alloy coating with at least 67% copper content on frequently touched surfaces. In fact, surgical instruments, resting chairs, handrails, and door handles in metros in Japan already have a copper alloy finish. Ideally, products that bear the Cu+ mark should be specified because they can eliminate viruses.

Prof. Anil Dewan added that, material finishes should be joint free and smooth, avoid using carpets and ceiling acoustic tiles.

### Reduction in the Airborne Transmission of Viruses in Healthcare

Infection prevention strategies should be more effective in public indoor spaces as depicted during the COVID-19 pandemic. Exceptional ventilation solutions and air purification systems must be implemented to avoid the transmission of airborne infections (Salonen et al., 2023).

Prof. Dr. Chandrashekhar R. noted that the technological methods used to reduce airborne transmission are pressurization dilution, filtration, and purification. Isolation rooms must have negative pressure to reduce the spread of infection, whereas operating theaters must have positive pressure to reduce the entry of viruses. The hospital should have anterooms before the negative pressure area, entry to the operating theater, and separate corridors for the movement of staff and patients.

He continued that, natural ventilation increases air changes per hour, which reduces the spread of infections. In addition, the use of prefilters, micro-eve filters, and high-efficiency particulate air (HEPA) filters can filter the air of pathogens, whereas ultraviolet germicidal irradiation can purify the air in the ducts. A central air cleaner is intended to provide purification for air-conditioning systems and can be customized to meet specific requirements. This is called the trap and kill technology. Meanwhile, free-standing air stabilization units are used for burn patients to protect against pathogens.

Prof. Anil Dewan remarked that ultraviolet germicidal irradiation with high-efficiency particulate air filters are necessary for infection control. Prof. Chandrashekhar pointed out that robotics uses ultraviolet rays that destroy viruses and can be used in wards, transportation facilities, and other areas of hospitals to sterilize spaces for new patients.

The audience questioned the sustainability of these design strategies. Prof. Chandrashekhar responded that ideally, there should be single-patient rooms for infection control. Prof. Chandrashekhar further explained that this increases the number of staff required to supervise patients. Hence, open nursing stations can monitor patients while using drapes between beds for division. In addition, the preferred method for controlling infections in hospitals is natural ventilation. However, HEPA filters are recommended in the design guidelines, but they should be carefully disposed of to avoid spreading contamination. Also, the Indian Green Building Council has developed guidelines in which they have identified the segregation of patients, asymptomatic, noninfected, and infected needing oxygen support to facilitate remote hospitals to reduce the spread of infections in India where such expertise is not available.

### Role of Digital Technology During the Pandemic

The use of digital technology to impart support, medical appointments, healthcare services, and track the spread of the COVID-19 virus has been recognized as an imperative solution to reduce the transmission of the pathogen (Alghamdi & Alghamdi, 2022).

Prof. Dr. Chandrashekhar observed that:

The shift to digital technology in hospitals was sped up by the COVID-19 pandemic, as this allowed the screening of patients. The AIIMS Hospital in Delhi experiences 10,000–12,000 footfalls in the outpatient department, and digital technology helped manage the crowd. Therefore, India introduced wellness centers to deal with less serious ailments, which accommodate multipurpose workers (male and female) and are led by a Mid-Level Health Provider, who screens patients and prioritizes those who need to go to the hospital.

### Indoor Environmental Quality and Healthy Buildings

Improved environmental quality can increase the value of the structure, improve the quality of occupants’ life, and increase productivity (Mujeebu, 2019).

Mr. Sanjay Prakash said the architectural office Shift, in India, has worked out how, for a single-story center, cross-ventilation can be achieved either with a depression of the corridor ceiling and providing ventilators or, for 4% extra area, the layout becomes single loaded around a courtyard, improving patient satisfaction.

Understanding the temperature setting in neonatal care in hospitals is imperative because infants are sensitive to temperature changes (Perlman & Kjaer, 2016). In fact, the temperature setting recommended by IPHS is 28 °C ± 2 °C in the neonatal care round the clock. The temperature inside special newborn care units (SNCU) should be set at the level of comfort (22 °C–25 °C) for the staff so that they can work for long hours. Unfortunately, air conditioning systems are optimized for temperatures of 22 °C–25 °C, not 28 °C ± 2 °C, thereby making it impossible to achieve that lower temperature by natural ventilation and light alone.

Mr. Deependra Prashad, Architect at DPAP Architects, noted that the sick building syndrome has been less addressed than energy and water consumption. In fact, the building can serve as a petri dish for various pathogens, whereas inadequate light causes vitamin D deficiencies. In response, a school in Meerut, India, has a brick facade, which is a breathable material that reduces moisture and sick building syndrome. Moreover, solar shading and limited openings control the admittance of sunlight. Furthermore, a trailer section of 12 feet allows hot air to rise. Meanwhile, Indira Parasaran Bhagwan in New Delhi has a five-star green rating from the IGBC. It has a shaded building envelope through recessed windows and a projecting solar top. Notably, the insulated walls and glass create a protected building envelope to minimize heat ingress along with operable windows to supplement the air-conditioning. However, the air must be filtered to reduce dust and pollutants. Moreover, the office is divided into zones to reduce the spread of pathogens.

He mentioned that the Uttar Haryana Bijli Vitran Nigam Limited head office building in Panchkula, India, uses ultraviolet germicidal irradiation to control viruses and is coupled with fine filters. However, the operable window is maintained to reduce the concentration of viruses. In addition, economizers can be used for energy efficiency by precooling air and avoiding the mixing of infected air with incoming air. The windows are open to air on the north and south sides for sunlight and cross ventilation. The window area is 15% of the floor area. Moreover, large-scale urban air quality can be controlled using urban water sprays, dust filters, and large green zones.

### Healthy Hospitals of Tomorrow

Prof. Dr. Anil Dewan defined a hospital as not just a place of therapeutic knowledge, research and technological innovation but also a place where professional and human relationships are activated. In design, we need a threefold model that prevents health-acquired infections by a design that protects, develops, and restores.

He recommended a horseshoe layout with separate entry, segregated and isolated buildings for resilience against COVID-19. In addition, he suggested that the first point of care should be with a rural or tribal facility.

### Evidence-Based Design in Operation Theaters

Evidence-based design principles use information from research when designing to improve productivity, clinical outcomes, economic performance, customer satisfaction, and cultural measures (Hamilton, 2003).

Dr. Anjali Joseph, the director of the Center for Health Design and faculty at Clemson University, noted that evidence-based design is a process for making decisions based on research to achieve the best outcomes. In fact, the architects’ designs could reduce patient stay and medication, reduce the number of patient falls, medical errors, and health-related infections. In fact, health-acquired infections affect one in 31 patients and can be spread by waterborne, airborne, and contact transmission. In response, the Dublin Hospital, United States, used lighting fixtures and aesthetic finishes for hand washing and located the sink in a visible location to entice users to use the sink.

Dr. Joseph retorted that the number of visits to surgical rooms has increased in the United States. Hence, it is important to segregate movement and reduce the distance traveled to minimize accidents and fatigue of the healthcare staff. The research conducted by Dr. Joseph used video observation, focus groups, and interviews to understand design challenges in the operating room. The design process used a cardboard model and simulations to test the solutions to the issues in the operating theater highlighted from the observations. The final design addressed the circulation of doctors, the location of storage, and the segregation of movement in the operating theater. The team designed a human-centered operating theater at the Emory Executive Park Musculoskeletal Institute in the United States. The audience asked why there were high surgical-acquired infections in the operating room despite the separate air-handling unit and antibacterial finishes. Dr. Joseph responded that the high number of doctors and healthcare staff working in operating theaters makes them prone to the spread of infection. Hence, the flow, including the storage location, is important to the operating theater.

### Evidence-Based Design in Practice

Dr. Upali Nanda, Global Practice Director, HKS Architects and faculty at University of Michigan, pointed out that, in the emergency pediatric department, the environment can reduce the risk of harm and anxiety because dysfunctional behavior among pediatric patients is less addressed. Footfall in this area can be reduced by creating zones and locating stores away from sterile areas.

In addition, the design strategies should address fall rates, medical errors, health-acquired infections, and time restraints and improve staff safety. Therefore, well-being in hospitals can be encouraged by service line stacking, shared family and staff zones, point of care supplies, sunrooms, and collaboration rooms. In addition, there can be a family lounge for patient satisfaction, a centralized nursing station, and a narrow core to reduce the walking distance of the staff. In fact, the team conducted research on the optimum location of the toilet and concluded that a nested toilet improves and resolves the issues of patient visibility, privacy, adequate family space, and views to the outside.

The work at the architectural firm HKS in the United States for researching hospital design efficiency involved shadowing, behavior mapping, noise-level measurements, focus groups, and interviews to develop the research. In addition, the HKS researchers documented the movement of stakeholders. For instance, nurses’ movements were monitored to determine the effect of changes in the layout. The design had a large footprint, but the efficient design reduced the walking distance by 43%. Nonetheless, the 55% expectation was not fulfilled, but there was a 30% noise reduction. On the other hand, the analysis of way-finding showed collaboration zones that were not used. However, these spaces were preferred for standardization, while sunrooms are ideal, glare was a problem, and there was a lack of dedicated areas for recreation. The speaker noted that design intent is lost in the design process; therefore, an operational handbook is necessary for architects. The audience asked how the hospital is designed for different cultures. Dr. Upali Nanda said that there are spaces specific to religion, more space for families, and climatic considerations that apply to each design. However, operable windows are adopted in Africa but are difficult to implement in the United States because of the climatic conditions. In addition, there is research on breathable facades for airflow. Finally, Prof. Anil asked, what were the changes after COVID-19 in the design of hospitals? Dr. Nanda replied “isolation rooms were located, separate elevators were installed, and the hospital took acute cases to reduce footfall.”

### Commercial Buildings

Climate change has changed crop yield patterns and sped up the development of pathogens (Priyadarsini et al., 2020). Hence, it is imperative to implement design strategies that reduce energy use and the spread of infections. Mr. Sanjit Roy, Assistant Professor at the School of Planning and Architecture, South Illinois University noted that:

The 200 West Street offices have communal spaces that encourage interaction. However, the position of the computer screens blocked natural daylight in the buildings. Meanwhile, the San Francisco Federal Building has skip stop elevators that stop at every other level. This encourages exercise and reduces energy consumption.

However, Dr. Prabhjot Sugga claimed that the aforementioned technologies may be difficult to implement in India due to cost implications. Mr. Sanjit Roy responded that the narrow building form is a simple strategy that can encourage natural ventilation and light. A good example is the School of Planning and Architecture in New Delhi, which has a narrow form that admits light and ventilation. In addition, Deependra Prashad, an architect at DPAP Architects, noted that active chilled beams are a healthier option because they reduce the need for air conditioning and save energy.

### Housing

Several mental and physical problems have become more prominent because of insufficient passive ventilation, natural daylight, and lack of green space in and around traditional high-rise residential buildings (Saadatjoo et al., 2023). In response, Prof. Manoj Mathur, Professor at the School of Planning and Architecture, New Delhi, stated the following:

Design guidelines have been introduced to create healthy homes and sanitary conditions. Although working from home is beneficial for infection control, the design should encourage outdoor interaction to reduce isolation and mental health issues. Admitting natural daylight reduces the need for artificial lighting while maintaining the circadian rhythm and increasing vitamin D levels. In addition, greenery is vital in residential areas, but at night, it is a source of harmful carbon dioxide. For this reason, in old Delhi, trees were not present near sleeping quarters. The concept of planting trees may have originated in the suburbs of developed countries, where homes are located at low density. Hence, the integration of trees into residential areas is imperative, but conducting case studies of successful integration is imperative before design. Dr. Prabhjot Sugga recommended gated cities as a design option that minimizes social isolation.

Meanwhile, Mr. Sanjit Roy noted that the design of Villa Savoye by Le Corbusier used long windows and flat roofs with gardens. On the other hand, the Frankfurt kitchen in Margarete (1926), during the Bauhaus movement, used clean metal tiles for flooring and was a highlight of modernism.

**Figure 1.**
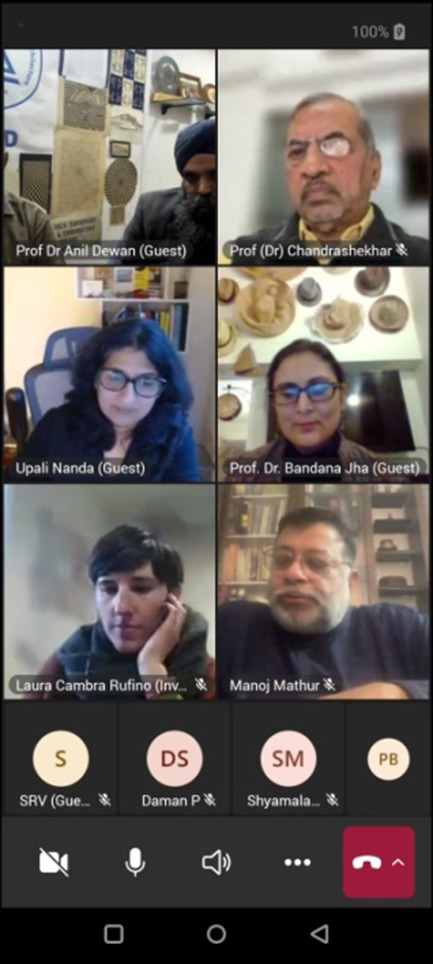
Screenshot of the focus group.

### Focus Group

The 35-min focus group discussion addressed the question “What were the changes in the design of hospitals post- pandemic?” (view Figure 1 for some of the panelists).

During the COVID-19 pandemic, in the Indian government hospitals, there was faster acceptance and implementation of digital technology. In fact, the implementation of telemedicine, presought appointments, and health cards helped reduce footfall and enabled reaching out to more ethnicities. Notably, the areas prone to infections during the COVID-19 pandemic were diagnostic and advanced operation areas. In response, the government’s Ayushman Bharat Scheme implemented separate COVID-19 blocks in all district hospitals and medical colleges. In addition, the government mandated oxygen plants in all hospitals. In addition, to encourage connectivity between hospitals, the new AIIMS hospitals have partnered with district/medical colleges for specialist treatments. Moreover, the use of digital systems is encouraged, as future scenarios are planned, developing countries can follow the example of Western countries and prepare for another infectious disease. However, India has advanced in digitization and climate responsiveness, which can be used to fight the pandemic. For instance, India had Aarogya Setu, a digital service for tracking COVID-19, implemented on a large scale. The Indian society adheres to sustainable practices, does not waste much, and prefers a link to nature; hence, retrofitting existing hospitals may be cost effective.

In fact, the pandemic provided an opportunity to use passive design for natural ventilation and lighting. Therefore, energy costs and the demand for oxygen are reduced. The Indian government district hospitals are naturally ventilated and use hybrid modes for air conditioning. A highly controlled environment is a foreign concept; instead, climate responsiveness and indoor environmental quality are prioritized. However, fresh air in the city environment is scarce.

Meanwhile, in Spain, overworking and excessive workload for healthcare workers was stressing the workforce, leading to several strikes.

There are seven lessons from the United States for pandemic resilience: (1) versatility and flexibility; (2) scenario planning for airborne diseases and terrorist attacks; (3) surge readiness, which is the ability to isolate and contain; (4) clean air and surfaces; (5) supporting well-being as infections impact families; (6) understanding circulation to minimize transmission risk ; and (7) implementation of digital and physical flexibility through telemedicine. These are huge lessons from the COVID-19 pandemic.

## Conclusion

**Figure 2.**
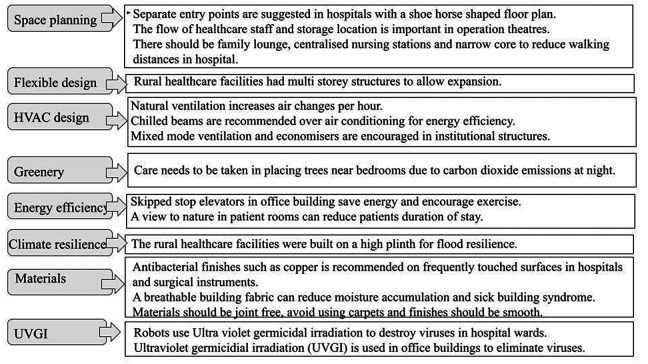
Highlights of plenary discussion by panelists. *Source*: Authors.

The Healthy Buildings webinar, organized by the School of Planning and Architecture in New Delhi on November 30, 2022, highlighted several design aspects that were highlighted during the pandemics (Figure 2). This information can help architects make wiser design decisions that encourage well-being and prepare buildings for the next infectious outbreak.

The webinar proved to be a useful platform for exchanging information from research projects and studies in the United States and India. The focus group discussion at the end generated ideas on how a hospital can be made resilient to future infectious disease outbreaks.
